# Quantitative analysis of mRNA expression levels and DNA methylation profiles of three neighboring genes: *FUS1*, *NPRL2/G21* and *RASSF1A* in non-small cell lung cancer patients

**DOI:** 10.1186/s12931-015-0230-6

**Published:** 2015-06-26

**Authors:** Dorota Pastuszak-Lewandoska, Jacek Kordiak, Monika Migdalska-Sęk, Karolina H. Czarnecka, Adam Antczak, Paweł Górski, Ewa Nawrot, Justyna M. Kiszałkiewicz, Daria Domańska, Ewa Brzeziańska-Lasota

**Affiliations:** Department of Molecular Bases of Medicine, Medical University of Lodz, Pomorska 251, C-5, 92-213 Lodz, Poland; Department of Chest Surgery, General and Oncological Surgery, University Hospital No. 2, Medical University of Lodz, Lodz, Poland; Department of General and Oncological Pulmonology, Medical University of Lodz, Lodz, Poland; Department of Pneumology and Allergology, Medical University of Lodz, Lodz, Poland

**Keywords:** Non-small cell lung cancer, Gene expression, Promoter methylation, Tumor suppressor gene, DNA methyltransferase

## Abstract

**Background:**

Tumor suppressor gene (TSG) inactivation plays a crucial role in carcinogenesis. *FUS1, NPRL2/G21* and *RASSF1A* are TSGs from LUCA region at 3p21.3, a critical chromosomal region in lung cancer development. The aim of the study was to analyze and compare the expression levels of these 3 TSGs in NSCLC, as well as in macroscopically unchanged lung tissue surrounding the primary lesion, and to look for the possible epigenetic mechanism of TSG inactivation *via* gene promoter methylation.

**Methods:**

Expression levels of 3 TSGs and 2 DNA methyltransferases, DNMT1 and DNMT3B, were assessed using real-time PCR method (qPCR) in 59 primary non-small cell lung tumors and the matched macroscopically unchanged lung tissue samples. Promoter methylation status of TSGs was analyzed using methylation-specific PCRs (MSP method) and Methylation Index (MI) value was calculated for each gene.

**Results:**

The expression of all three TSGs were significantly different between NSCLC subtypes: *RASSF1A* and *FUS1* expression levels were significantly lower in squamous cell carcinoma (SCC), and NPRL2/G21 in adenocarcinoma (AC). *RASSF1A* showed significantly lower expression in tumors *vs* macroscopically unchanged lung tissues. Methylation frequency was 38–76 %, depending on the gene. The highest MI value was found for *RASSF1A* (52 %) and the lowest for *NPRL2/G21* (5 %). The simultaneous decreased expression and methylation of at least one *RASSF1A* allele was observed in 71 % tumor samples. Inverse correlation between gene expression and promoter methylation was found for *FUS1* (rs = −0.41) in SCC subtype. Expression levels of DNMTs were significantly increased in 75–92 % NSCLCs and were significantly higher in tumors than in normal lung tissue. However, no correlation between mRNA expression levels of DNMTs and DNA methylation status of the studied TSGs was found.

**Conclusions:**

The results indicate the potential role of the studied TSGs in the differentiation of NSCLC histopathological subtypes. The significant differences in *RASSF1A* expression levels between NSCLC and macroscopically unchanged lung tissue highlight its possible diagnostic role in lung cancer *in situ* recognition. High percentage of lung tumor samples with simultaneous *RASSF1A* decreased expression and gene promoter methylation indicates its epigenetic silencing. However, DNMT overexpression doesn’t seem to be a critical determinate of its promoter hypermethylation.

## Background

The development of non-small cell lung cancer (NSCLC) is associated with molecular changes in more than 20 genes localized in different chromosomal regions. The most frequent molecular event in lung cancer pathogenesis is allele loss (loss of heterozygosity, LOH) on short arm of chromosome 3 (3p), in multiple, so called critical regions. LOH analyses in 3p indicate two frequently affected regions (FARs) within 3p21.3: LUCA (lung cancer region) in the centromeric region (3p21C) and AP20 (Alu-PCR clone 20 region) in the telomeric region (3p21T), which contain loci of multiple tumor suppressor genes (TSGs) [1]. Inactivation of TSGs is the key event in carcinogenesis and involves two steps, each of them affecting one allele. Most frequently, one TSG allele is lost due to LOH and the other – to mutation, or, alternatively, epigenetic inactivation *via* gene promoter hypermethylation. The studies on LOH analysis in lung cancer indicated that 3p allele loss is nearly universal, shows a “discontinuous LOH pattern” involving multiple, discrete sites, and appears to occur firstly in the 600-kb 3p21.3 region [2]. The reported incidence of LOH in LUCA region varied, ranging from 10 to 74 %, however, it was always higher in squamous cell carcinoma (SCC) than in adenocarcinoma (AC), up to 96 % and 50 %, respectively [1–3]. Additionally, low mutation rate, below 10 %, found in lung cell lines and primary tumors, highlighted the significance of epigenetic modifications of TSGs in lung carcinogenesis [4, 5].

Epigenetic mechanisms, which include DNA methylation, post translational modifications to core histones, microRNA (miRNA) and long non-codingRNA (lncRNA) regulation [6], play a crucial role in regulation of gene expression by affecting chromatin accessibility. The best known epigenetic modification in human is DNA methylation. Cancer-related aberrant DNA methylation pattern, e.g., hypermethylation of promoter sequences of TSGs, provide a range of opportunities for risk assessment, early detection, disease progression and prognosis, as well as therapeutic stratification and post-therapeutic monitoring of cancer. In lung carcinogenesis, among the potential DNA methylation biomarkers, the hypermethylation of *APC*, *CDH1*, *CDH13*, *DAPK1*, *FHIT*, *MGMT*, *p16INK4a*, *RARβ, RASSF1A, RUNX3* and *SHOX2* is the most frequently reported [5, 7, 8]. However, among the ever increasing number of new studies there are also those that negate the previous conclusions [9].

DNA methylation is an enzymatic process mediated by DNA methyltransferases (DNMTs). DNMT1, possessing a 7- to 21- fold preference for hemimethylated DNA than unmethylated DNA, is the primary enzyme responsible for copping methylation patterns, i.e., for its maintenance [10]. DNMT3 family enzymes (−3A and -3B) exhibit *de novo* methylation activity, as they have similar affinities for both unmethylated and hemi-methylated DNA substrates, and affect the methylation status of normally unmethylated CpG sites [10]. Additionally, a cooperation between DNMT3 family and DNMT1 has been shown in cancer [11]. DNMTs not only play a pivotal role in carcinogenesis, being responsible for DNA methylation, but also seem to be promising molecular bio-markers for cancer diagnosis and therapy [12].

The pre-specified hypothesis tested in the study was that the expression levels of 3 selected TSGs from LUCA region (*FUS1, NPRL2/G21* and *RASSF1A*) were decreased in primary non-small cell lung cancer with promoter hypermethylation as the responsible epigenetic mechanism of their silencing. To investigate the factors involved in TSG hypermethylation in NSCLC, we attempted to determine whether *DNMT1* and *DNMT3B* RNA expression levels correlated with the hypermethylation of the promoters of the studied tumor suppressor genes. We tried to elucidate the role of the studied TSGs in early lung carcinogenesis and cancer progression.

## Materials and methods

The study has been approved by the Ethical Committee of the Medical University of Lodz, Poland, no. RNN/140/10/KE. Written informed consent was obtained from each patient.

### Characterization of the lung tissue samples and patients clinical characteristics

The study involved the group of 59 patients with diagnosis of NSCLC, treated in the University Clinic of Pneumology and Allergology of I^st^ Chair of Internal Diseases of Medical University of Lodz and in the Department of Thoracic Surgery, General and Oncologic Surgery, Medical University of Lodz, Poland, between July 2010 – March 2013. In NSCLC patients, during the planned surgery (lobectomy or pneumonectomy), tissue fragments (100–150 mg) were obtained from the center of primary lesion and the adjacent noncancerous (10 cm distant from the primary lesion), macroscopically unchanged tissue (conventional “normal” sample). Immediately after resection, lung tissue samples were placed in a stabilization buffer RNAlater®. Each tissue sample was divided into smaller parts (30–50 mg) for individual analysis, and frozen at −80 °C.

The resected tissue samples were post-operatively histhopathologically evaluated and classified according to the AJCC staging as well as TNM classification (pTNM). Based on histopathological assessments, the group of patients was subdivided in relation to NSCLC subtypes, i.e., squamous cell carcinoma (SCC), adenocarcinoma (AC) and large cell carcinoma (LCC). Relevant clinical and pathological characteristics of the patients with NSCLC included in this study are summarized in Table 1. All cases were primary tumors without chemo- or radiotherapy treatment.

**Table 1:** Clinicopathological features of the studied NSCLC group

Analyzed variables	NSCLC patients	
Gender (n, %)	Women (24, 41 %)	
	Men (35, 59 %)	
Age (n, %)	Mean 61 ± 7.62	≤60 yrs (14, 24 %)
		61-70 yrs (30, 51 %)
		>70 yrs (15, 25 %)
Smoking status and smoking history (n, %)	Smokers (54, 92 %)	<40 Pack Years^a^ (PYs) (26, 48 %)
	current smokers (31, 57 %)	
	former smokers (23, 43 %)	≥40 PYs (28, 52 %)
	non-smokers (5, 8 %)	
Histopathological type of NSCLC (n, %)	SCC (34, 58 %)	
	NSCC (25, 42 %)	AC (20, 80 %)
		LCC (5, 20 %)
AJCC classification^b^ (n, %)	I (11, 18 %)	
	II (21, 36 %)	
	III (27, 46 %)	
Tumor size according to pTNM classification^c^ (n, %)	T1 (12, 20 %)	
	T2 (33, 56 %)	
	T3/T4 (14, 24 %)	

^a^PYs were calculated according to the NCI Dictionary of Cancer Terms: 1 Pack Year is equal to 20 cigarettes smoked per day for 1 year (http://www.cancer.gov/dictionary?CdrID=306)

^b^AJCC – American Joint Committee on Cancer Staging according to the IASCLC Staging Project 7th ed. (2010) Cancer

^c^pTNM – post-operative Tumor Node Metastasis classification according to the WHO Histological Typing of Lung Tumor

The design of our study assumed the analysis of gene expression in two study groups: the tumor and the surrounding normal lung tissue, to compare the both type of tissue. The more so, because there are reports indicating no significant differences between cancerous and non-cancerous tissue in regard to some TSG expression [13]. On the other hand, accumulating evidence point to genetic/epigenetic changes occurring in normal tissue adjacent to tumor (the so called “field cancerization” process), which are widely described in literature in various cancer types and highlights the importance of early abnormalities in carcinogenesis [14, 15].

### RNA extraction, real-time PCR (qPCR method)

Total RNA was extracted from primary lung cancer and macroscopically unchanged lung tissues, using Universal RNA Purification Kit (Eurx, Poland), according to the manufacturer’s recommendations. The qualitative and quantitative assessments of RNA samples were determined using RNA 6000 Pico/Nano LabChip kit (Agilent Technologies, USA) in Agilent 2100 Bioanalyzer (Agilent, USA).

Complementary DNA (cDNA) was transcribed from 100 ng of total RNA, using a High-Capacity cDNA Reverse Transcription Kit (Applied Biosystems, USA). Reverse transcription (RT) master mix contained: 10x RT buffer, 25x dNTP Mix (100 mM), 10x RT Random Primers, MultiScribe™ Reverse Transcriptase, RNase Inhibitor and nuclease-free water, in a total volume of 20 μl. RT reactions were performed in the following conditions: 10 min at 25 °C, 120 min at 37 °C, then 5 s at 85 °C for, and 4 °C hold.

The relative expression of the studied genes was assessed in qPCRs using Micro Fluidic Cards, the so called TLDA (TaqMan® Low Density Custom Arrays) plates, with pre-loaded selected assays (Applied Biosystems, USA): *FUS1*-Hs00200725_m1, *NPRL2/G21*-Hs00198012_m1, *RASSF1A*-Hs00200394_m1, *DNMT1*-Hs00945875_m1, *DNMT3B*-Hs00171876_m1. *ESD* (Hs00382667_m1) was used as a reference gene and RNA isolated from normal lung tissue (Human Lung Total RNA, Ambion®, USA) served as calibrator samples.

All qPCRs were performed using 7900HT Fast Real-Time PCR System (Applied Biosystems, USA) with RQ software (TaqMan Relative Quantification Assay software). The reaction mixture contained: 50 μl cDNA (50 ng) and 50 μl TaqMan® Universal Master Mix (Applied Biosystems, USA). The PCR program was as follows: initial incubation 2 min at 50 °C, AmpliTaq Gold® DNA polymerase activation at 94.5 °C for 10 min, then 40 two-step cycles 30 s at 97 °C and 60 s at 59.7 °C.

### DNA extraction, bisulfite conversion and methylation-specific PCRs (MSP method)

The extraction of genomic DNA from NSCLC specimens was performed using a QIAamp DNA Mini Kit (Qiagen, Germany), according to the manufacturer’s protocol. To obtain RNA-free genomic DNA, RNase A (100 mg/ml) was used as optionally indicated in the kit procedure. The quality and quantity of DNA was spectrophotometrically assessed, measuring absorbance at 260/280 nm (Eppendorf BioPhotometer™ plus, Germany). DNA samples with a 260/280 nm ratio in the range 1.8–2.0 were considered as high quality and selected for further analysis.

Genomic DNA (1 μg) was modified with sodium bisulfite, using the CpGenomeTM Turbo Bisulfide Modification Kit (CHEMICON International, Millipore, USA), according to the manufacturer’s protocol. Its concentration and purity was spectrophotometrically estimated (Eppendorf BioPhotometer™ plus, Germany).

Bisulfite converted DNA was used for methylation-specific polymerase chain reactions (MSPs) to assess methylation status of the studied genes. Primers for MSPs were designed according to the criteria described by Feltus et al. [16], using computer tool (methPrimer v1.1 beta, Li Lab, Department of Urology, USCF) [17]. The set of primers for the studied genes were flanking the 1 kb 5′ region upstream from the translation start point Primer sequences for methylated and unmethylated promoter regions of the studied genes are included in Table 2. MSP master mix contained: 1000 ng DNA, 0.7 μM of each primer (Sigma-Aldrich, Germany), 2.5 μM dNTPs mix, 2.5 μM MgCl_2_, Hot Start AmpliTaq Gold® 360 DNA Polymerase (5U/μl), 10x Universal PCR buffer and nuclease-free water, in a total volume of 12.5 μl. PCR conditions were as follows: initial denaturation at 95 °C for 5 min, followed by 40 three-step cycles involving denaturation at 95 °C for 45 s, specific annealing temperature – (see Table 2) – for 45 s and elongation at 72 °C for 1 min; the final elongation step was done at 72 °C for 10 min. The annealing temperatures for each MSP primer were experimentally determined in a set of gradient PCRs. The range of temperatures tested, covering these given in individual primer specifications (Sigma-Aldrich, Germany) were as follows: 50.5–60.5 °C for *FUS1*, 52.5–64.2 °C for *NPRL2/G21*, and 53–66.5 °C for *RASSF1A*.

**Table 2:** Characterization of MSP primers (M – methylated; U – unmethylated) used in the study. The underlined nucleotides in forward and reverse primers indicate the presence of methylated cytosines in DNA sequence

Gene	Forward primer	Primer position	Reverse primer	Primer position	Product length [bp]	Annealing temp. [°C]
*FUS1*	M: TGTTATCGTGGATTAGATATTGTTC	−559 to −482	M: ACTATATTTTTACGATTACCACGCT	−375 to −349	207	56.5
	U: TTATTGTGGATTAGATATTGTTTGT	−557 to −480	U: ACTATATTTTTACAATTACCACACT	−375 to −349	205	56.5
*NPRL2/G21*	M: GTTCGGTTATTGTTATGGGTAGC	+108 to +131	M: AACCAATTAAACTCTCGAAAACGT	+238 to +261	153	52.5
	U: GGTTTGGTTATTGTTATGGGTAGTG	+107 to +132	U: ACCAATTAAACTCTCAAAAACATC	+238 to +261	153	56.5
*RASSF1A*	M: ATATTTTTTCGATTTGGAGTTTTTTC	−599 to −573	M: CTACACTATAACCTACCCATCCTCG	−409 to – 384	215	56.5
	U: TTTTTTTGATTTGGAGTTTTTTTGT	−599 to −573	U: TACACTATAACCTACCCATCCTCAC	−410 to −385	211	57.5

Positive and negative MSP controls were included in each PCR reaction. CpGenome Universal Methylated DNA (enzymatically methylated human male genomic DNA) served as a positive methylation control and CpGenome Universal Unmethylated DNA (human fetal cell line) was used as a negative control (CHEMICON International, Millipore, USA). As a control for PCR contamination, blank samples with nuclease-free water instead of DNA were used.

The MSP products were electrophoretically separated on 2 % agarose gel and concentrations (ng) of MSP products (U and M DNA alleles) were spectrophotometrically estimated, using DNA1000 LabChip Kit, on Agilent 2100 Bioanalyzer (Agilent Technologies, USA). For each sample, Methylation Index (MI) was assessed using the following formula: peak height of methylated products/(peak height of methylated products + peak height of unmethylated product), MI = (M)/(M + U).

### Statistical analysis

The results of relative expression analysis (RQ values) are presented as medians in each studied group. The comparison of RQ values between cancer and non-cancer specimens was performed using Mann–Whitney *U* test and Kruskal-Wallis test. The same nonparametric tests were applied to compare the differential expressions and methylation status of TSGs between NSCLC subtypes, i.e., SCC, AC and LCC. Spearman’s rank correlation coefficient, Mann–Whitney *U* test and Kruskal-Wallis test were performed to evaluate the relationship between gene expression and methylation levels and clinicopathological parameters (patients’ characteristics: age, gender, history of smoking and tumor staging according to pTNM and AJCC classifications). The Newman–Keuls method was used to identify significantly different samples, regarding gene expression and methylation levels. Nonparametric Spearman’s criterion was used to calculate the coefficient of correlation between the levels of mRNA expression or promoter methylation for pairs of studied TSGs and in pairs with methyltransferases. Receiver operating characteristic (ROC) curve was established to discriminate NSCLC and adjacent normal tissue.

*P*-values < 0.05 were considered statistically significant. All statistical procedures were performed using Statistica for Windows 10.0 software.

## Results

### Relative expression levels of the studied TSGs

Relative expression levels of the studied tumor suppressor genes in NSCLC and macroscopically unchanged lung tissue samples were determined using delta-delta C_T_ method, and expressed as RQ values adjusted to the expression of *ESD* (endogenous control) and in relation to the expression level of calibrator (normal lung tissue), for which RQ = 1.

**Fig. 1:**
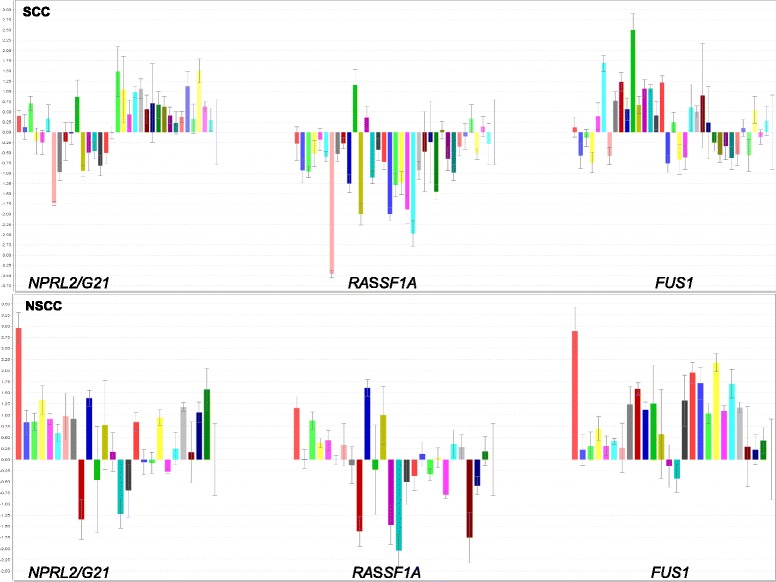
Relative expression levels of the three TSGs, presented as log_2_RQ, in the studied NSCLC histopathological subtypes

The obtained RQ values, for the studied TSGs in individual NSCLC samples, are presented in Fig. 1. The simultaneous down regulation of all 3 TSGs was found only in 5.9 % specimens. Simultaneous decreased expression of *RASSF1A* and *FUS1* was observed in 23.5 % SCC samples, and paired *RASSF1A* and *NPRL2/G21* were simultaneously decreased in 20.9 % SCC and 16 % NSCC samples. Generally, *RASSF1A* gene showed decreased expression in the majority of lung tumors (especially in SCC subtype), while the expression levels of *NPRL2/G21* and *FUS1* in most cases were similar to the level of calibrator. Table 3 shows the frequency of NSCLC samples with importantly decreased expression.

**Table 3:** Frequency of importantly decreased TSG expression (RQ < 0.5)

Gene	NSCLC samples (T)			Paired “normal” samples (N)		
	Total	SCC	NSCC	Total	SCC	NSCC
	(*n* = 59)	(*n* = 34)	(*n* = 25)	(*n* = 59)	(*n* = 34)	(*n* = 25)
*RASSF1A*	23.7 %	29.4 %	16 %	5.1 %	2.9 %	8 %
	(*n =* 14)	(*n =* 10)	(*n =* 4)	(*n =* 3)	(*n =* 1)	(*n =* 2)
*NPRL2/G21*	5.1 %	2.9 %	8 %	3.4 %	2.9 %	4 %
	(*n =* 3)	(*n =* 1)	(*n =* 2)	(*n =* 2)	(*n =* 1)	(*n =* 1)
*FUS1*	0 %	0 %	0 %	1.7 %	2.9 %	0 %
	(*n =* 0)	(*n =* 0)	(*n =* 0)	(*n =* 1)	(*n =* 1)	(*n =* 0)

The obtained RQ values were correlated with histopathological NSCLC subtypes (SCC, AC, LCC), tumor staging (pTNM, AJCC), patients’ age, gender and smoking history, as well as with RQ values of the studied genes in macroscopically unchanged lung tissue samples. Additionally, correlations between all studied genes were analyzed.

Comparing NSCLC histolopathological subtypes, the expression levels of *RASSF1A* and *FUS1* were significantly different, while in case of *NPRL2/G21* gene, only a tendency was observed. Regarding patients’ age, *NPRL2/G21* showed significantly lower expression in older patients. Concerning patients’ sex, *RASSF1A* showed significantly lower expression level in females in NSCC group, and significantly lower expression in males in SCC group. In relation to tumor size (pTNM classification), *RASSF1A* expression was the lowest in T2 group in total NSCLC group, and similarly in NSCC subgroup. Regarding AJCC classification, statistically significant differences were observed for *FUS1* gene in NSCC subtype. Comparing tumor and macroscopically unchanged lung tissue samples, statistically significant differences were found for all genes, however only in case of *RASSF1A* gene its expression level was significantly lower in tumors. No significant differences were observed in correlation with cigarette smoking. The results are summarized in Table 4.

**Table 4:** Correlations analyzed between the expression levels of the studied genes and tumor clinicopathological parameters and patients’ characteristics

*FUS1* (*n =* 59)	*NPRL2/G21* (*n =* 59)	*RASSF1A* (*n =* 59)
Median RQ in NSCLC samples		
1.35	1.36	0.77
SCC *vs* AC *vs* LCC		
1.18 *vs* 1.83 *vs* 2.39 P = 0.002^a^	1.28 *vs* 1.79 *vs* 0.83 P = 0.05^a^	0.68 *vs* 1.01 *vs* 0.86 P = 0.01^a^
SCC *vs* AC		
1.18 *vs* 1.83 P = 0.004^b^	1.28 *vs* 1.84 P = 0.03^b^	0.68 *vs* 1.01 P = 0.002^b^
SCC *vs* NSCC		
1.18 *vs* 2.13 P = 0.001^b^	*P* > 0.05	0.68 *vs* 1.01 P = 0.002^b^
Age (≤60 yrs *vs* 61–70 yrs *vs* >70 yrs) – in total NSCLC group; in NSCLC histopathological subgroups		
*P* > 0.05	In SCC: 61–70 yrs *vs* >70 yrs	*P* > 0.05
	1.55 *vs* 0.99 P = 0.001^b^	
Gender (females *vs* males) – in total NSCLC group; in NSCLC histopathological subgroups		
*P* > 0.05	*P* > 0.05	In SCC: 1.04 *vs* 0.64 P = 0.02^b^;
		in AC: 0.86 *vs* 1.31 P = 0.01^b^;
		in NSCC: 0.86 *vs* 1.31 P = 0.02^b^
pTNM classification (T1 *vs* T2 *vs* T3/4) – in total NSCLC group; in NSCLC histopathological subgroups		
*P* > 0.05	*P* > 0.05	0.80 *vs* 0.67 *vs* 0.94 P = 0.04^a^;
		T2 *vs* T3/4 P = 0.01^b^;
		in NSCC: 1.35 *vs* 0.75 *vs* 1.27 P = 0.04^a^; T2 *vs* T3/4 P = 0.04^b^
AJCC classification (I *vs* II *vs* III) – in total NSCLC group; in NSCLC histopathological subgroups		
In NSCC: 2.44 *vs* 1.20 *vs* 2.25 P = 0.04^a^; I *vs* II P = 0.02^b^	*P* > 0.05	*P* > 0.05
Smoking status (current smokers *vs* former smokers *vs* never smokers) – in total NSCLC group; in NSCLC histopathological subgroups		
*P* > 0.05	*P* > 0.05	*P* > 0.05
Smoking history (PYs: <40 *vs* ≥40) – in total NSCLC group; in NSCLC histopathological subgroups		
*P* > 0.05	*P* > 0.05	*P* > 0.05
Tumor (T) tissue *vs* macroscopically unchanged lung tissue – in total NSCLC group; in NSCLC histopathological subgroups		
in AC: 1.83 *vs* 1.18 P = 0.01^b^;	1.36 *vs* 0.84 P = 0.0003^b^;	0.77 *vs* 1.07 P = 0.0003^b^;
in NSCC: 2.13 *vs* 1.20 P = 0.002^b^	in SCC: 1.28 *vs* 0.84 P = 0.01^b^;	in SCC: 0.68 *vs* 1.09 P = 0.000002^b^
	in AC: 1.84 *vs* 0.84 P = 0.002^b^;	
	in NSCC: 1.79 *vs* 0.84 P = 0.009^b^	

^a^Kruskal-Wallis test; ^b^Mann–Whitney *U*-test

Newman-Keuls test documented statistical significances between the expression levels of the studied genes in pairs in NSCLC group, as well as in NSCLC histopathological subtypes: statistically significant differences were found between *RASSF1A* vs *NPRL2/G21* (P = 0.001) and *RASSF1A* vs *FUS1* (P = 0.00002) in total study group, and similarly in SCC subtype, where significant differences were found between *RASSF1A* vs *NPRL2/G21* (P = 0.001) and *RASSF1A* vs *FUS1* (P = 0.0003); in AC and NSCC subtypes, the expression levels of *RASSF1A* and *FUS1A* were significantly different (P = 0.03 and P = 0.005, respectively). In all mentioned cases, *RASSF1A* expression level was significantly lower than those of the other genes. Spearman’s rank correlation coefficient revealed positive correlations between genes: *RASSF1A* and *NPRL2/G21* (P = 0.0001, rs = 0.38), *RASSF1A* and *FUS1* (P = 0.001, rs = 0.34) in NSCLC group; and also between *RASSF1A* and *NPRL2/G21* in AC (P = 0.02, rs = 0.52) and NSCC (P = 0.002, rs = 0.60) group.

ROC curve analysis of *RASSF1A* expression level as a marker to distinguish NSCLC tissue from macroscopically unchanged lung tissue demonstrated a sensitivity of 61 %, specificity of 73 % and accuracy of 67 % (AUC = 0.695). Additionally, we analyzed the utility of *RASSF1A* and *FUS1* as potential markers distinguishing NSCLC histopathological subtypes: NSCC and SCC. Analyzed together, the genes yielded a sensitivity of 69 %, specificity of 66 % and accuracy of 68 % (AUC = 0.714). Separately, ROC curve analysis illustrated for *RASSF1A*: a sensitivity of 79 %, specificity of 64 % and accuracy of 73 % (AUC = 0.729); and for *FUS1*: a sensitivity of 88 %, specificity of 56 % and accuracy of 74 % (AUC = 0.729).

### Relative expression levels of the studied DNMTs

Relative expression of two DNA methyltransferases, DNMT1 and DNMT3b, were also analyzed on mRNA level. The expression levels of both DNMTs were increased in tumor samples: median RQ value for *DNMT3B* was 3.81 and for *DNMT1* 1.38. Regarding the frequency of samples with increased *DNMT* expression, *DNMT1* was overexpressed in 75 % NSCLCs (68 % SCC, 76 % NSCC) and *DNMT3B* in 92 % NSCLCs (91 % SCC, 92 % NSCC).

Correlations were analyzed between the expression levels of the studied *DNMT*s and histopathological NSCLC subtypes (SCC, AC, LCC), tumor staging (pTNM, AJCC), patients’ age, gender and smoking history, as well as in relation to RQ values of the studied *DNMT*s found in macroscopically unchanged lung tissue samples. *DNMT3B* expression level was significantly higher in younger patients (≤60 *vs* 61–70 years old) in AC subtype (7.14 *vs* 2.43, P = 0.04, Mann–Whitney *U*-test) and in NSCC subtype (7.37 *vs* 2.43, P = 0.006, Mann–Whitney *U*-test), where also the negative correlation was observed (rs = −0.50, P = 0.01, Spearman’s rank correlation coefficient). No statistically significant differences were found in relation to patients’ gender and smoking, or tumor size (pTNM classification) and grade (AJCC classification). However, in SCC group *DNMT1* revealed a trend (P = 0.05, Mann–Whitney *U*-test) toward higher expression in grade I/II vs grade III tumors (1.53 vs 1.10).

The expression levels of both DNMTs were significantly higher in tumors when compared with macroscopically unchanged lung tissue samples, in total NSCLC group and in NSCLC histopathological subgroups, as shown in Table 5.

**Table 5:** RQ values (medians) correlated between tumor *vs* macroscopically unchanged lung tissue samples, with *P* value (Mann–Whitney *U*-test)

Gene	total NSCLC group	SCC subgroup	AC subgroup	NSCC subgroup
*DNMT1*	1.38 *vs* 0.71 P = 0.0001	1.36 *vs* 0.69 P = 0.0001	1.57 *vs* 0.74 P = 0.0001	1.38 *vs* 0.73 P = 0.0002
*DNMT3B*	3.81 *vs* 1.63 P = 0.0001	5.24 *vs* 1.61 P = 0.0001	3.63 *vs* 1.72 P = 0.002	3.57 *vs* 1.64 P = 0.001

Additionally, the expression levels of the studied DNA methyltransferases were positively correlated (Spearman’s rank correlation coefficient) in all tissue groups: in total NSCLC cohort (rs = 0.51, P = 0.0001), in SCC (rs = 0.51, P = 0.002), in AC (rs = 0.49, P = 0.03), in NSCC (rs = 0.55, P = 0.004).

There weren’t any negative correlations between the levels of expression of the methyltransferases and TSGs.

### Methylation status of the studied TSG genes: the qualitative and quantitative assessments

The results on methylation status of the studied genes were obtained for different numbers of patients, due to DNA degradation in several samples: 17 in case of *RASSF1A*, 3 in cases of *NPRL2/G21* and *FUS1*.

Promoter methylation analysis of the studied genes, performed as MSP reactions, distinguished unmethylated (U) from methylated (M) DNA alleles after electrophoretic separation. Both U and M alleles were found in all studied tumor tissue groups. Next, based on spectrophotometric estimation (Agilent 2100 Bioanalyzer), fluorescence units (FU) of MSP products were quantified (ng/μl), according to DNA size marker (DNA ladder, Agilent Technologies, USA) and Methylation Index (MI) was calculated for each gene in all tissue samples.

**Fig. 2:**
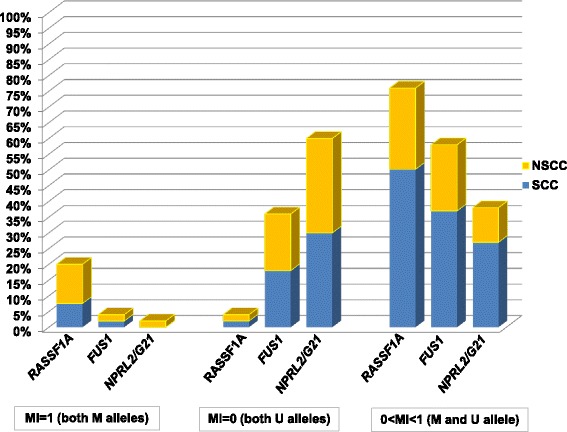
The frequencies of methylated (M) and unmethylated (U) alleles of the studied genes, based on MI values

The MSP reactions revealed the presence of M alleles for all genes, in 38–76 % samples, depending on the gene. The frequencies of methylated and unmethylated alleles of all studied TSGs, are shown in Fig. 2. The highest frequency of both methylated alleles was observed for *RASSF1A* gene (20 %) and the same gene revealed the lowest number of samples with no methylated alleles (4 %). Generally, the most abundant were samples with the presence of both methylated and unmethylated alleles (38–76 %, depending on the gene).

The calculated MIs indicated gene methylation level, which in tumor samples ranged from 3 to 66 %, depending on the gene. Among the studied genes, *RASSF1A* showed the highest methylation level: 52 % in total NSCLC group, 50 % in SCC, and 66 % in AC; *NPRL2/G21* – the lowest: 5 % in total NSCLC group, 3 % in SCC, and 10 % in AC. Methylation level of *FUS1* was 17 % in total NSCLC group, also 17 % in SCC and 10 % in AC.

**Fig. 3:**
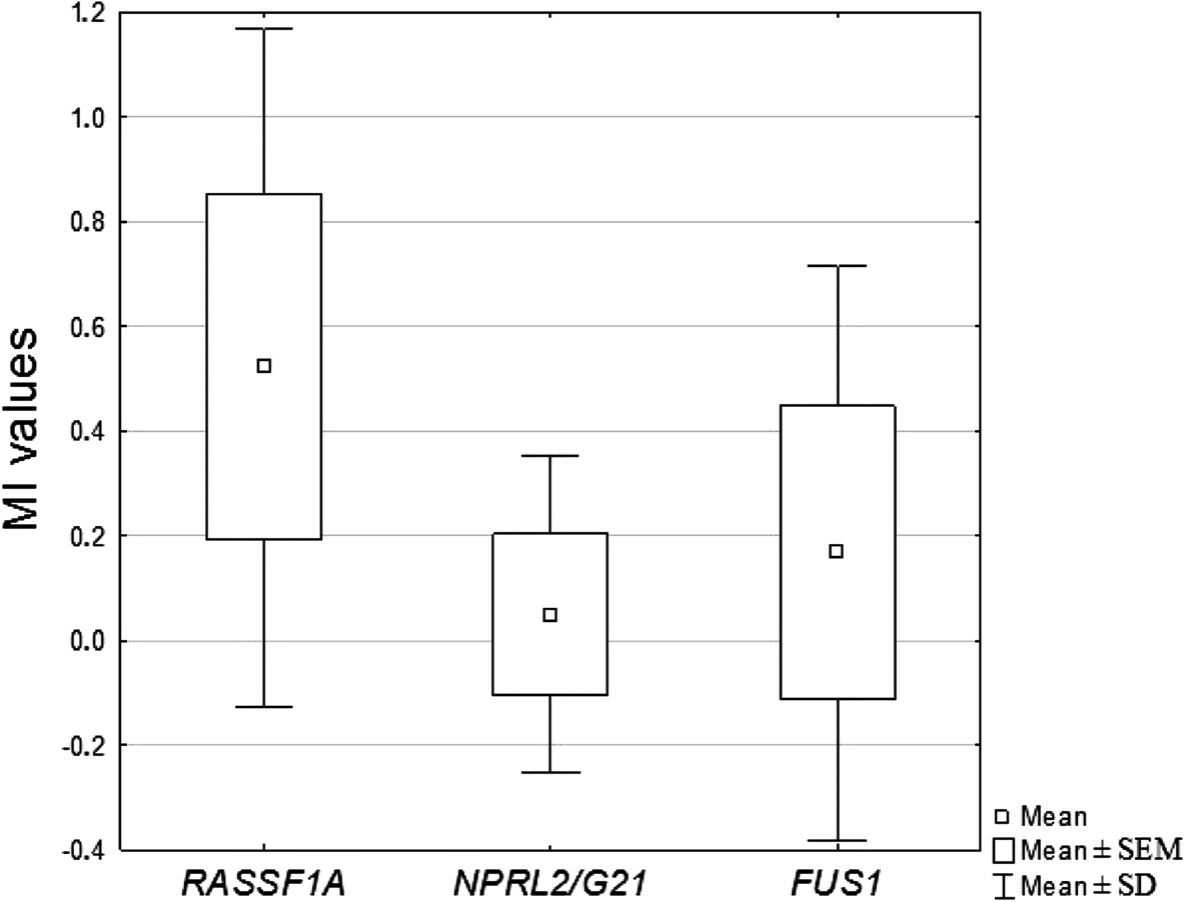
Box and whisker plot representing mean MI values of the studied genes in NSCLC group

Statistical analysis (Newman-Keuls test) revealed significantly higher methylation status of *RASSF1A* than those of other studied genes in all tissue groups. In NSCLC: *RASSF1A vs FUS1* (P = 0.0001), *RASSF1A vs NPRL2/G21* (P = 0.00002); MI values of *FUS1* were also significantly higher than *NPRL2* methylation status (P = 0.01) in this tissue group. The results are shown in Fig. 3. Similarly in SCC group: *RASSF1A vs NPRL2/G21* (P = 0.00002), *RASSF1A vs FUS1* (P = 0.0001); and also *FUS1 vs NPRL2/G21* (P = 0.04). In AC: *RASSF1A vs NPRL2/G21* (P = 0.0001), *RASSF1A vs FUS1* (P = 0.0001).

Concerning tumor histopathological characteristics, in SCC group *FUS1* was significantly higher methylated in stage II *vs* stage III tumors (P = 0.03, Mann–Whitney *U* test). No other significant correlations were found between gene methylation and clinicopathological parameters.

### Correlation between gene expression level and methylation status

The decreased expression of *RASSF1A* was accompanied by promoter methylation in 71 % NSCLC samples, in case of *FUS1* it was found in 20 % NSCLC specimens, and only in 5 % NSCLC samples for *NPRL2/G21*. Statistical analysis revealed the negative correlation between the RQ and MI values for only one gene, *FUS1* (P = 0.02, Spearman’s rank correlation coefficient, rs = −0.41) in SCC subtype.

Spearman’s rank correlation coefficient didn’t reveal statistically significant correlations between TSG methylation and DNMT expression (*P* > 0.05).

## Discussion

The aim of our study was to analyze by qPCR and compare the expression levels of 3p TSGs in non-small cell lung cancer samples, as well as in macroscopically unchanged tissue surrounding the primary lesion. To look for the possible epigenetic mechanism of TSG inactivation, gene promoter methylation was assessed, as well as the levels of DNMT1 and DNMT3B RNA expression.

To the best of our knowledge, only few studies were performed to compare expression of several 3p TSGs in the same lung tumor samples [18–22]. In order to investigate it further we chose 3 TSGs and analyzed their expression in lung primary tumors: non-small cell lung cancer (NSCLC), adenocarcinoma (AC), squamous cell lung cancer (SCC) and large cell cancer (LCC). Similarly, few studies have focused so far on simultaneous analysis of methylation status of more than two genes at 3p21.3 locus in primary lung cancer [21, 23, 24].

The chosen TSGs, namely *FUS1*, *NPRL2/G21* and *RASSF1A*, are located in proximity, in 3p21.3 subregion called LUCA. Such close position could suggest the tendency toward simultaneous decrease of their expression. However, this was not the case in our study. Only few tumor samples showed decreased expression of all three genes. The low levels of promoter methylation of NPRL2/G21 and, to some extent, of FUS1 also didn’t designate their epigenetic inactivation in the population studied.

The highest frequency of decreased expression and increased promoter methylation we found for *RASSF1A* gene. The simultaneous decreased expression and methylation of at least one *RASSF1A* allele was found in 71 % tumor samples. Tumor suppressor gene, *RASSF1A* (*Ras association domain family member 1*), is involved in development or progression in the vast majority of cancers [4, 25, 26]. In NSCLC, *RASSF1A* decreased expression was found in 67 % samples, as reported by Senchenko et al. [21], although it is usually reported at the level of about 30 % [27]. In our study we confirmed decreased expression of *RASSF1A* (RQ < 1), and the important gene downregulation (RQ < 0.5) was observed in more than 20 % of NSCLC samples, with higher frequency in SCC group (reaching nearly 30 %). In paired “normal” lung tissue specimens *RASSF1A* decreased expression was found only in 3–8 % samples. The differences between the studied groups, i.e., between NSCLC subtypes and between primary tumor specimens and the surrounding macroscopically unchanged lung tissue samples, were statistically significant (in total study group and in SCC subtype). The highest *RASSF1A* expression level – similar to that observed in “normal” lung tissue – was found in AC group. Similarly, the increased level of *RASSF1A* mRNA in lung cancer was also observed by others, who found even 3-to-4 fold increase in AC subtype [21]. *RASSF1A* gene – as the only one in our analysis – revealed correlations with gender: significantly lower gene expression was found in men in SCC group and in women in NSCC group. Regarding TNM classification, significantly lower *RASSF1A* expression was observed in T2 tumors as compared with T3/4 NSCLC. It could confirm its role at earlier stages of lung carcinogenesis. However, in other studies, no statistically significant differences were observed with relation to age, smoking history and other cytological and pathological characteristics [21].

Significant differences found between tumor and “normal” lung samples indicate the possible clinical utility of *RASSF1A* gene expression level as a diagnostic marker. In most studies, the principal cause of *RASSF1A* loss of expression was tumor-specific *RASSF1A* promoter hypermethylation, while tumor-associated mutations were rare [25, 26, 28]. In our study, the frequency of *RASSF1A* methylated alleles in total NSCLC group was 76 % and gene methylation level was equal to or exceeded 50 %, depending on the histotype. The observed methylation frequency of *RASSF1A* was in the top of the reported by others 26–87 % range [23, 25, 29–34].

In several reports, *RASSF1A* methylation was associated with poor prognosis in NSCLC patients [30–32, 35]. We didn’t observe any associations with tumor clinicopathological features, such as TNM or AJCC staging, or patients’ smoking habits. Similar results were obtained by others [34, 35]. Meta-analysis performed in 2013 confirmed a significant association between *RASSF1A* methylation and NSCLC, however there were no significant differences in *RASSF1A* methylation in relation to gender, pathology, TNM stage and smoking behavior among NSCLC cases [36].

It is true that of all the genes in the 3p21.3 critical region *RASSF1* has been the most comprehensively studied at the genetic, epigenetic and functional level, and its role as a tumor suppressor gene has been proven. However, the results regarding the utility of *RASSF1A* epigenetic inactivation as a prognostic marker in NSCLC are still divergent [9, 27, 31, 33]. In our study, *RASSF1A* methylation level was significantly higher than that of other studied genes, however, lack of any other associations does not entitle us to decisive conclusions.

Another gene localized in LUCA region, *FUS1* (*fused in sarcoma*), also known as *TUSC2* (*tumor suppressor candidate 2*) is recently identified TSG, involved in apoptosis. Loss or reduction of FUS1 protein expression, associated with poorer prognosis, was observed in lung cancers [37–39]. We didn’t observe importantly decreased expression of *FUS1* on mRNA level in lung tumor samples. Statistically significant differences between NSCLC histotypes and the lowest gene expression level in SCC might suggest *FUS1* significance predominantly in squamous cell carcinoma. In NSCC subtype, significantly decreased *FUS1*expression in stage II *vs* stage III tumors could indicate its role at early stage of carcinogenesis. However, generally, *FUS1* expression level was similar to that observed for calibrator, i.e., normal lung tissue. No aberrant *FUS1* mRNA level was also found by other researchers [28]. *FUS1* promoter methylation analysis in our study revealed the presence of methylated alleles in 58 % NSCLC samples and rather moderate gene methylation status, ranging from 10 to 22 %, depending on histopathological subtype. However, as the only gene in our analysis, *FUS1* revealed negative correlation between its expression level and methylation status. Additionally, significantly higher gene promoter methylation level in II *vs* III tumors in SCC subgroup might support *FUS1* possible role at early stage of lung cancer development.

So far, there have not been published any reports on *FUS1* promoter hypermethylation in lung cancer, although it was found to be partially methylated in head and neck squamous cell carcinoma [40]. Little is known on molecular mechanisms involved in the regulation of *FUS1* expression in primary lung cancer cells. *FUS1* mRNA transcripts were found in both normal lung and some lung cancer cell lines, but FUS1 protein was absent in a majority of lung cancer cell lines and NSCLC samples [38]. Researchers indicate the role of 3'-untranslated regions (UTRs) of the *FUS1* gene transcript [41] or the influence of another epigenetic mechanisms, e.g., the role of several miRNAs in the down-regulation of FUS1 protein expression in lung cancer cells [42] or aberrant histone modifications [43]. Thus, the inactivation of FUS1 may occur through abnormal translational control of *FUS1* mRNA or aberrant post-translational protein modification resulting in its inactivation despite the absence of mutations and in the presence of high levels of transcript mRNA [44].

The physiological role of FUS1 is still poorly known and further investigation of this interesting TSG in lung cancer is required.

*NPRL2/G21* (*nitrogen permease regulator-like 2 gene*), also known as *TUSC4* (tumour-suppressor candidate 4), is a tumor suppressor gene commonly expressed in normal tissues, including lung tissue. The study results indicate that *NPRL2/G21* is involved in DNA repair, cell cycle control and apoptosis [45]. Senchenko et al. [21] showed significantly decreased gene expression level in 73 % NSCLC cases, but the earlier report described normal *NPRL2/21* mRNA expression in most lung cancers [25]. Stop mutations, nonsense deletions and missense mutations were found in this gene [18, 45]. Epigenetic analyses failed to show gene promoter hypermethylation in cancer cell lines [43, 45], and no such studies have been performed so far in primary lung tumors.

In our study, we didn’t confirm decreased expression of *NPRL2/G21* in lung tumor samples. However, the differences between NSCLC histopathological subtypes were significant, with lower expression level in SCC samples. Although, it was still at the level of calibrator (normal lung tissue). In some studies, increased expression of NPRL2/G21 on mRNA or protein level was also observed [21, 46]. Interestingly, *NPRL2/G21* gene – as the only TSG in our study group – showed correlation with age: in SCC samples its expression level was significantly lower in elder patients (>70 years old).

The exact mechanism involved in the possible inactivation of *NPRL2/G21* in human cancers has not been elucidated. In our study we didn’t observe hypermethylation of *NPRL2/G21* promoter region. The frequency of gene methylation in NSCLC samples was very low and its methylation status was significantly lower than that of other studied TSGs.

According to the results obtained by Lerman & Minna [28], *NPRL2/G21* may be one of the haploinsufficient genes that predispose to cancer in a hemizygous state and do not show a second mutation – or promoter hypermethylation – in the other allele (wild-type allele). Alternatively, as in the case of other 3p21.3 tumor suppressors, like *RASSF1A* and *FUS1*, such mechanisms as chromosome instability, aneuploidy, altered RNA splicing, or defects in transcriptional, translational, and posttranslational modifications that are common in the 3p region, may play a role in the inactivation of *NPRL1/G21* in lung tumors.

Although the function of *NPRL2/G21* is still unknown, it revealed interesting association in lung cancer: gene decreased expression was conversely correlated with cisplatin sensitivity in NSCLC cell lines [46]. This finding, of significant clinical value, could indicate the possible therapeutic role of *NPRL2/G21* for enhancing and re-sensitizing non-responders to cisplatin. Therefore, *NPRL2*/*G21* deserves attention and further study in lung cancers, regarding its expression and underlying mechanisms of its possible silencing.

None of the genes revealed correlations with smoking history. It is in accordance with the results of Senchenko et al. [21]. Although smoking is a known risk factor in NSCLC, the lack of correlations with expression levels and methylation status of the studied TSGs could indicate that tobacco smoke targets other than studied genes.

We tested whether the methylation status of tumor suppressor genes was associated with the mRNA expression levels of DNA methyltransferases (DNMTs). The role of DNMT mediated epigenetic alterations in lung cancer development has been the focus of increasing interest in recent years. In our study both enzymes were highly expressed in a coordinate manner in lung tumors – positive correlation was observed in all NSCLC histopathological types, as well as in total NSCLC group. In case of both studied DNMTs, we found significantly higher expression levels in lung tumor specimens as compared with macroscopically unchanged tissue samples.

Regarding NSCLC histopathological subtypes, *DNMT3B* expression was higher in SCC than in NSCC, although it didn’t reach statistical significance. *DNMT1* revealed trend toward higher expression in grade I/II tumors in SCC group, suggesting its role at early stages of lung carcinogenesis. However, in other studies, deregulated expression of *DNMT1* was an independent prognostic factor in SCC [47, 48]. Interestingly, in AC and NSCC subtypes, *DNMT3B* expression levels were significantly higher in patients aged 60 and below as compared with older patients. Elevated DNMT expression in younger patients was more frequent also in other study [48].

We didn’t observe any negative correlations between the expression levels of the studied TSGs and methyltransferases. The results of others are also conflicting. Similarly to our findings, the elevated mRNA levels of *DNMT1* and *DNMT3B* were not significantly associated with hypermethylation of the several TSGs, including *RASSF1A*, in lung cancer cell lines [49] and in lung primary tumors [48, 50]. Others found correlations between TSG promoter hypermethylation and DNMT overexpression, but on protein level [47]. Overexpression of *DNMT* was found to occur earlier than the methylation modifications and complex interactions between several factors in hypermethylation process was highlighted [48, 50]. So, there is still a need to elucidate the clinicopathological significance of DNA methyltransferases in primary non-small cell lung cancer.

## Conclusions

The results of our study indicate the potential role of the studied genes in the differentiation of NSCLC histopathological subtypes: *RASSF1A* and *FUS1* could be regarded as markers differentiating SCC and NSCC, as their expression levels were significantly lower in squamous cell carcinoma subtype; similar role could be assign to *NPRL2/G21* in differentiating SCC and AC subtypes. Although, the most prominent role was documented in case of *RASSF1A*. The significant differences in its expression level between NSCLC and macroscopically unchanged lung tissue surrounding the primary lesions highlight its possible diagnostic role in lung cancer *in situ* recognition. High percentage of lung tumor samples with simultaneous *RASSF1A* decreased expression and gene promoter methylation indicates its epigenetic silencing. However, overexpression of methylating enzymes (DNMT1 and DNMT3B) enzymes was not a critical determinate of tumor-specific promoter hypermethylation of *RASSF1A* and *FUS1*, which revealed, respectively, high and moderate methylation frequency in our study.
